# Evaluating a Tobacco-free Policy Intervention in Residential Substance Use Disorder Programs

**DOI:** 10.1177/00220426251322707

**Published:** 2025-02-24

**Authors:** Anna Pagano, Caravella McCuistian, J. Konadu Fokuo, Jennifer Le, Joseph Guydish

**Affiliations:** 1 Department of Psychiatry and Behavioral Sciences, University of California, San Francisco, CA, USA; 2 Neuropsychiatric Institute, 14681 University of Illinois, Chicago, IL, USA; 3 Institute for Health Policy Studies, 8785 University of California, San Francisco, CA, USA

**Keywords:** tobacco cessation, tobacco intervention, tobacco-free grounds, implementation, qualitative, SUD treatment

## Abstract

**Introduction:** Individuals in treatment for substance use disorders (SUD) use tobacco at much higher rates than the general population. Qualitative research can help identify successful implementation approaches for tobacco-free policy interventions in SUD treatment, but qualitative post-intervention evaluation studies are limited. **Methods:** Directors of sixteen residential SUD treatment programs participated in a multi-year tobacco-free policy intervention. Semi-structured interviews (*n* = 32) were conducted at 12- and 18 months post-intervention. Interview transcripts were analyzed thematically to determine implementation barriers and supports. **Results:** Supports for tobacco-free policies included a broader wellness context, alternatives to smoking, community partnerships, gradual change, and patient inclusion. Barriers were patient cessation challenges, staff resistance, and insufficient capacity. **Conclusion:** Implementation of tobacco-free policies in residential SUD treatment programs is feasible but requires significant planning and support. Single state agencies and other substance use treatment authorities should allocate more resources to support implementation of tobacco-free policies in SUD treatment.

## Introduction

Patients in treatment for substance use disorders (SUD) smoke at very high rates (Baldassarri et al., 2019; Min et al., 2022) and are more likely to die from tobacco-related causes than from alcohol or other drug use (Bandiera et al., 2015; Callaghan et al., 2018). Tobacco use is associated with increased risk of SUD relapse (Weinberger et al., 2017), while tobacco cessation is associated with improved SUD treatment outcomes (Tsoh et al., 2011).

Tobacco-free policy interventions in SUD treatment programs have been shown to decrease tobacco use among patients (Gubner et al., 2019; Guydish et al., 2017; McCuistian et al., 2022). Tobacco-free policy interventions may include both environmental (e.g., tobacco-free grounds) and clinical (e.g., assessment and treatment/referral for smoking cessation) components. These components may be mutually reinforcing, since patients in tobacco-free programs are more likely to receive clinical services for tobacco cessation (Guydish et al., 2019).

Although SUD treatment programs are increasingly adopting tobacco-free policies, implementation remains limited outside of major hospital and treatment systems (Marynak et al., 2018). In a national sample of SUD treatment providers in the United States, 52% of outpatient facilities and 68% of residential facilities permitted smoking in designated outdoor areas (Holt et al., 2024). Major implementation barriers include lack of financial resources and workforce expertise (Knudsen, 2017). Moreover, staff and patients often believe that quitting tobacco use during SUD treatment will cause relapse to other substances (Pagano et al., 2016). Treatment programs may also fear a drop in patient census if they implement tobacco-free grounds, although this has proven unfounded (Richey et al., 2017). While some studies have pinpointed factors such as increased staff training to support implementation of tobacco-free policies in SUD treatment programs (Campbell et al., 2022), less is known about other factors that may influence the implementation of these policies.

In 2018, the California Tobacco Control Program created the Tobacco-Free for Recovery (TFR) initiative to help residential SUD programs implement tobacco-free and other wellness policies. The initiative provided financial and technical support to increase workforce expertise regarding tobacco cessation policies and treatment. By addressing these major implementation barriers (financial and technical), the initiative aimed to increase the implementation of tobacco-free policy interventions. The current study provides qualitative post-intervention data on barriers and supports for tobacco-free policies, as reported by program directors in sixteen SUD programs participating in TFR.

Prior qualitative reports concerning tobacco-related interventions in SUD treatment have focused on general perceptions or attitudes rather than evaluating specific interventions (Gentry et al., 2017; Iyahen et al., 2023). Qualitative studies of pre-intervention barriers and supports for tobacco-free policies identified low stakeholder engagement, lack of workforce expertise, and lack of reimbursement for tobacco cessation services as barriers; and program directors’ commitment to planning and engaging policy champions as supports (Fokuo et al., 2022; Pagano et al., 2016). However, there is limited qualitative data on the experience of programs after they have participated in a tobacco-free policy initiative.

The current qualitative study was conducted as part of a larger study featuring both qualitative and quantitative components in a pre-post intervention design. Quantitative findings from surveys with program staff and patients are reported elsewhere (McCuistian et al., 2022, 2024). The present report follows a pre-intervention analysis of program director interviews from the same programs participating in the TFR initiative (Fokuo et al., 2022). For the data reported here, we conducted 12- and 18-month post-intervention interviews with directors of programs participating in TFR to identify elements of effective approaches to implementing tobacco policy, as well as strategies used to surmount barriers and support sustainability.

## Methods

The TFR initiative was a multi-year program designed to encourage tobacco cessation in residential behavioral health programs across California (CTCP, 2018). The Smoking Cessation Leadership Center (SCLC) (Vijayaraghavan et al., 2024) provided technical assistance to help programs design tobacco-free policies and provide tobacco cessation services. The initiative also required programs to create wellness policies promoting other healthy lifestyle changes among clients (e.g., physical activity, improved nutrition). Analyses showed that the initiative was associated with decreased smoking prevalence among patients (McCuistian et al., 2022).

### Program Selection and Recruitment

The California Tobacco Control Program disseminated notice of the TFR initiative through its partnership network in California. Licensed residential behavioral health treatment programs with a minimum 15-bed capacity were eligible for participation. Sixteen eligible SUD programs were selected and received incentives over three years to offset costs. Selected programs had 24- to 185-bed capacities.

### Policy Intervention

The TFR initiative supported programs in developing, implementing, or strengthening policies that: 1) reduced or prohibited tobacco use on program grounds (i.e., a tobacco-free grounds policy); 2) established procedures for assessing and treating tobacco use among patients; and 3) incorporated wellness activities to support a tobacco-free environment (e.g., using former smoking areas for physical activity). While programs were required to design a tobacco-free grounds policy, they were not required to implement the policy during the initiative.

To implement these goals, participating programs took the following actions: (1) evaluate existing tobacco-related policies and complete needs assessments for tobacco-free policy implementation; (2) meet monthly with the Smoking Cessation Leadership Center to develop tobacco-related policies and report progress; and (3) participate in quarterly learning community meetings with leads from all participating programs to discuss experiences and share lessons learned. The Smoking Cessation Leadership Center provided policy and implementation plan templates for tobacco-free grounds, facilitated external consultation on wellness policy development, and provided tobacco cessation training to clinical staff.

### Data Collection

We purposively sampled and conducted semi-structured interviews with directors of 16 programs at 12- and 18-month intervals following the intervention. Our final sample included 18 directors, since some programs included more than one director. The 16 programs entered the initiative in cohorts starting at different times, so that follow-up interviews were completed between November 2019 and May 2022.

Interviews, lasting about 1 hour, were conducted via Zoom. The interview guide was informed by the Consolidated Framework for Implementation Research (Damschroder et al., 2022) and included questions related to the following domains: procedures for creating and disseminating policies to staff and patients; current tobacco-related policies and procedures; barriers and supports for implementing tobacco-related policies and procedures; directors’ attitudes about tobacco cessation during SUD treatment; current tobacco use treatment approaches; and reimbursement for tobacco cessation services. Interviewers were trained in qualitative interviewing, research ethics, and human subjects protection. Interviews were digitally recorded and professionally transcribed. Interviewees received a $50 gift card for their time.

Seventeen interviewees (one director from 15 programs; two directors from one program) also completed an online survey including their demographic characteristics (i.e., age, gender, race/ethnicity, smoking status, personal SUD recovery status, and number of years working at the SUD treatment program) and their programs’ tobacco-free policies (e.g., tobacco-free grounds policy, rules concerning patient and staff smoking, and tobacco cessation services provided). In a few cases of missing data, we consulted online profiles and program websites to estimate data.

All participant information was de-identified, and study procedures were approved by the Institutional Review Board of the University of California, San Francisco.

### Data Analysis

Descriptive statistics were calculated for interviewee demographic data. These included mean and standard deviation for the count variable (age), and frequencies and percentages for categorical variables (gender, race/ethnicity, smoking status, SUD recovery status, years of experience). Analysis of policy surveys consisted of simple binary counts (yes/no to indicate if a policy was implemented at the 12- or 18-month time point) and frequencies for number of tobacco-free policies at each time point per program.

Qualitative interview data were analyzed using thematic analysis techniques (Boyatzis, 1998) and coded using ATLAS.ti (Muhr, 2013). Aside from the a priori themes of “implementation barriers” and “implementation supports,” the coding framework emerged organically from the data. Through repeated review and open coding of the data, a trained qualitative analyst (AP) identified salient themes and devised a taxonomy to represent thematic hierarchies. Theme salience was assessed using two criteria: (1) frequency with which the theme recurred *across the interview sample*, and (2) the degree of emphasis placed on that theme by an interviewee *within a given interview* (Buetow, 2010). Degree of emphasis was determined by the relative proportion of text devoted to a given theme within an interview, as well as the relative importance of the theme to the study’s research questions.

Once major themes were identified, a coding taxonomy was developed and entered into ATLAS.ti, then applied electronically to relevant interview text. Next, the analyst selected interview passages to exemplify major themes and salient subthemes, presented below. In excerpted passages, “P#” indicates the number assigned to each program for confidentiality, and “# months” indicates whether the interview occurred at 12 or 18 months after the program began to participate in the initiative.

## Results

Respondent characteristics are summarized in Table 1, and Table 2 summarizes tobacco policy characteristics for each program at 12 and 18 months post-intervention. Policy characteristics included tobacco-free grounds, as well as policies intended to support movement toward tobacco-free grounds. These included: outdoor smoking not allowed for patients; outdoor smoking not allowed for staff; staff and patients not allowed to smoke together; no designated smoking breaks for patients; nicotine replacement therapy (NRT) and/or cessation medications available; and smoking cessation screening and/or counseling available.

**Table 1.** Participant Demographic Characteristics (*N* = 17).^a^

Characteristic	*n* (%)
Age (M, SD)	48.8 (11.8)
Female	15 (93.8)
Race/Ethnicity	
Non-Hispanic White	11 (68.8)
Non-Hispanic Black	2 (12.5)
American Indian/Alaska Native	2 (12.5)
Latino/Hispanic	1 (6.3)
Education	
Some college	5 (29.4)
Master’s degree	9 (52.9)
Other – professional licensing only	3 (17.6)
In recovery from substance use	11 (84.6)
Smoking status^**^	
Current smoker	1 (7.7)
Former smoker	8 (61.5)
Never smoker	6 (46.2)
Years at current SUD treatment program	
1–5 years	7 (43.8)
6–10 years	4 (25.0)
11–15 years	1 (6.3)
More than 15 years	4 (25.0)

^a^Of the 16 participating programs, 15 returned one survey and one returned two surveys.

**Denominators vary due to missing data.

**Table 2.** Tobacco-free Policies at 12 and 18 months Post-intervention in Residential SUD Programs.

12 Months post-intervention								
Prog. #	TFG^a^ at time of interview?	Outdoor smoking not allowed for patients	Outdoor smoking not allowed for staff	Staff/patients not allowed to smoke together	No designated smoking breaks for patients	NRT/Cessation medication available	Smoking cessation screening or counseling available	# of policies that support tobacco free grounds
1	Yes	Yes	Yes	Yes	X	X	Yes	4
2	X	X	X	X	X	Yes	Yes	2
3	Yes	Yes	Yes	Yes	Yes	X	Yes	5
4	X	X	X	X	Yes	Yes	Yes	3
5	Yes	Yes	Yes	Yes	Yes	Yes	Yes	6
6	X	X	X	Yes	X	Yes	Yes	3
7	Yes	Yes	Yes	Yes	Yes	X	Yes	3
8	X	X	X	Yes	X	Yes	Yes	3
9	X	X	X	Yes	Yes	Yes	Yes	4
10	X	X	X	Yes	Yes	X	Yes	3
11	X	X	Yes	Yes	X	Yes	Yes	4
12	X	X	Yes	Yes	X	Yes	Yes	4
13	Yes	Yes	Yes	Yes	X	Yes	Yes	5
14	X	X	X	Yes	Yes	Yes	Yes	4
15	TFG @ BL	Yes	Yes	Yes	Yes	Yes	Yes	6
16	TFG @ BL	Yes	Yes	Yes	Yes	Yes	Yes	6
18 Months post-intervention								
Prog. #	TFG^a^ at time of interview?	Outdoor smoking not allowed for patients	Outdoor smoking not allowed for staff	Staff/patients not allowed to smoke together	No designated smoking breaks for patients	NRT/Cessation medication available	Smoking cessation screening or counseling available	# of policies that support tobacco free grounds
1	Yes	Yes	Yes	Yes	Yes	X	Yes	5
2	Yes	Yes	Yes	Yes	Yes	Yes	Yes	6
3	Yes	Yes	Yes	Yes	Yes	X	Yes	5
4	X (Aft int.)	X	X	Yes	Yes	Yes	Yes	4
5	Yes	Yes	Yes	Yes	Yes	Yes	Yes	6
6	X	X	X	Yes	Yes	X	Yes	3
7	Yes	Yes	Yes	Yes	Yes	X	Yes	5
8	X	X	X	Yes	Yes	Yes	Yes	4
9	X	X	X	Yes	Yes	Yes	Yes	4
10	X (Aft. Int.)	X	X	Yes	Yes	Yes	Yes	4
11	X	X	Yes	Yes	X	Yes	Yes	4
12	X	X	Yes	Yes	X	Yes	Yes	4
13	Yes	Yes	Yes	Yes	X	Yes	Yes	5
14	X	X	X	Yes	Yes	Yes	Yes	4
15	TFG @ BL	Yes	Yes	Yes	Yes	Yes	Yes	6
16	TFG @ BL	Yes	Yes	Yes	Yes	Yes	Yes	6

^a^TFG = “Tobacco-free grounds.”

• Programs marked “Yes” adopted tobacco-free grounds during the TFR initiative. Those marked “X” did not.

• “Aft. int.” means the program adopted tobacco-free grounds during the TFR, but after the 18-month interview was conducted.

• “TFG @ BL = “Tobacco-free grounds at baseline” means the program already had tobacco-free grounds before the TFR initiative started.

At the time of the 12-month interview, all participating programs had at least one tobacco-free policy. At that point, seven of the 16 programs had fully implemented tobacco-free grounds. Of these, two (P15, P16) already had tobacco-free grounds at baseline, while the other four had adopted tobacco-free grounds during the intervention. Ten programs had other tobacco-related policies but did not yet have tobacco-free grounds.

At the time of the 18-month interview, one more program (P2) had fully implemented tobacco-free grounds for a total of eight out of 16. Among the remaining eight programs, three (P4, P8, P10) had increased the number of tobacco-free policies between the 12- and 18-month interviews. Two (P4, P10) went on to fully implement tobacco-free grounds after the 18-month interview.

### Implementation Supports

#### Wellness Context

The most frequently mentioned support at both 12 and 18 months was to approach tobacco-free policies as part of a broader wellness framework. Strategies included replacing “smoke breaks” with “wellness breaks” (P3) in which walking was encouraged rather than smoking; changing the program’s outdoor smoking area to a gardening area (P2); providing alternatives to smoke breaks such as yoga classes (P4, P8); and emphasizing mindfulness meditation and daily gardening for stress relief during tobacco cessation (P1, P8, P11). One interviewee described wellness-focused activities as both a starting point for tobacco cessation attempts and as longer-term maintenance tools:

I think if you have a program especially like ours where we … promote health and wellness, we foster the healthy eating, and we foster assistance with exercise, …we have yoga, we have meditation, we have a garden, so we have so many things to assist you to quit smoking. And then from there, we start talking about the stay quit, what you going to do when you get cravings, what are you going to have when those triggers, when the stressors of life come back, so for you not to go back to that. (P1, 18 months)

Another emphasized the value of mindfulness meditation techniques as a strategy to resist cravings while attempting to quit smoking:

So, now that they’re not using drugs - tobacco, you know, nicotine is still a drug, and so they’re using that as a substitute to their – to the illegal drugs. So, increasing their ability to use healthy coping skills through mindfulness, and the practice of it - it’s one thing to know it, but to practice it.…I really like it when the patients say, ‘Oh, [interviewee name], I used mindfulness today…and I was able to not react to that craving to have that cigarette. (P11, 18 months)

Some interviewees described a clustering effect whereby focusing on one health behavior increased the likelihood that patients would also focus on other health behaviors. For instance, one interviewee observed connections between patients’ attempts to stop smoking and simultaneous efforts to improve their physical activity or nutrition:

I do notice that as people work on one area in their health, that it kind of spreads out a little bit. You know, somebody who’s not smoking and they start walking maybe during their outside break for a little bit. Then that can lead to making better choices as far as what they eat or how much they eat and it kind of builds on itself. (P8, 18 months)

#### Alternatives to Smoking

Another frequently mentioned support was providing patients with alternatives to smoking. This subtheme was related to the “wellness context” subtheme since the alternatives often involved healthier activities, but it was distinct in that the focus was on distraction from smoking rather than on the activities themselves. Interviewees who expressed this subtheme also emphasized replacing the reward mechanism of nicotine use with other rewards.

So I had to… cut down on how many smoke breaks they get, things like that. But then I replace it with something because I can’t take a coping skill that they’re used to and not replace it with something. So usually it’s some incentive that they like; more phone time or here’s more exercise, here’s another yoga class, here’s more healthy type snacks that you can have throughout the day. (P8, 12 months)

Besides incentives such as extra privileges and breaks, other alternatives to smoking included activities such as gardening or physical exercise to distract patients and help them combat stress and cravings. One interviewee described outdoor and nature-focused activities as the most effective alternatives to smoking, especially during the first month of treatment when cravings were most intense:

Putting in the garden, having alternative activities for clients to participate in when they’re outdoors, having an area for them just to go and be and put their hands in the soil to work on has made a difference.…I bought all outdoor exercise equipment because we don’t really have space inside the facility. So, just the combination of bringing their awareness to doing these alternative activities with exercise and outdoor activity has already been a positive distraction, especially for the new clients… in the first 30 days it’s really important to have these kinds of things available because that’s when they are craving the cigarettes the most because they just got there, and they may have been a heavy smoker. (P15, 18 months)

#### Community Partnerships

Interviewees emphasized the importance of external partnerships to accomplishing their goals. In addition to financial and technical assistance from the grant partners, they cited group calls with other agencies participating in the policy initiative (P1, P6); joining tobacco control committees in the community (P6); asking the local public health department to provide nutrition and tobacco cessation educational groups (P10, P16); obtaining staff tobacco cessation training from local community clinics (P13); and obtaining assistance with NRT and other tobacco cessation medications from community clinics and hospital-based “bridge” clinics (P11, P13).

One interviewee reported that the ability to compare notes with other SUD programs participating in the TFR initiative was instrumental in their own implementation:

The first thing that was helpful were the group calls with other programs, share with other people, get ideas. We have the monthly calls with other agencies that have been so helpful. I also have the calls with [California Tobacco Control Program] to help me stay up to date with the requirements. I also see online what other people are doing that could work for us and our program, so overall it’s been helpful working with other people who are trying to adapt change along the way. (P1, 12 months)

Another interviewee stressed the importance of making community referrals for tobacco cessation medications available to patients from the start of the program. Some partnered informally with community clinics for that purpose:

…[W]hen someone comes in, we automatically ask, “do you smoke, do you need a referral, we’re smoke-free, how do you think you’ll be able to handle [it]?” We make a referral … If the client goes there and says like, hey, you know, I need to stop smoking, they prescribe like [varenicline]. (P13, 18 months)

#### Gradual Change

Several interviewees (P1, P2, P3, P5, P6, P8) noted that they faced less patient resistance to tobacco-free changes by introducing tobacco-free policies incrementally rather than all at once:

…[Patients] initially could smoke at any time in the designated smoking areas outside the building. So first we started by allowing them to smoke only during breaks, and we stopped calling them “smoke breaks” and started calling them “wellness breaks” and so we rebranded and then eventually went smoke free. It was definitely a process and maybe that’s why there wasn’t much resistance. (P3, 12 months)

Two interviewees described a gradual process of slowly cutting back on smoking areas and breaks:

…[W]e gave people notices: “There will be one less cigarette,” and at this point we had a designated outside smoking area. We told them beforehand we’re going to cut it in half. The area was going to get smaller and smaller, and then it won’t be there. So they visually saw stuff…even though there was some resistance we just kept moving through. (P2, 18 months)

So it went from smoking being open from 5am to 10pm, to smoking being open from 5am to 7pm for a couple of days…We kept pulling the hours back until…we were doing that call to action, when we were going to completely stop smoking, we were like “smoking only here for only 1 hour today,” and then it was gone. (P1, 18 months)

Interviewees who had moved to nonsmoking grounds during the TFR initiative emphasized the importance of preparing patients and staff ahead of time for the transition. This included providing not only NRT but also tobacco cessation counseling and psychoeducation before implementing a full smoking ban:

…NRT of course is always optional, but we let them know, “hey, we’re gonna be going tobacco-free, this would help you, it would also help you with recovery, blah blah blah,” so we put in a lot of the supports ahead of time, ahead of going tobacco-free, so we already had lots of physical activity, we had already made our nutrition changes. We had already implemented NRT…and systematically screening and discussing it with everybody who came through admissions and did their physical. So all those pieces were ahead of going tobacco-free. (P5, 12 months)

We had people who were available for them to talk to, if they needed to talk about cravings, about triggers, about the causative behavior, we got together smoking patches, you know, gums and toothpicks and so we kind of did a lot of research on our end. So we said hey we understand that is not something that is going to be easy but we are going to be here to support you. (P1, 18 months)

#### Patient Inclusion

Several interviewees (P3, P5, P6, P10) noted that patient inclusion in the implementation process was key to obtaining buy-in and mitigating resistance to tobacco-free policy changes. One interviewee’s (P6) staff conducted an initial meeting with patients to discuss an upcoming electronic cigarette ban (the program was already tobacco-free). When the patients expressed concerns, the executive team met with the patients to discuss ways to make the transition easier:

Both [the men’s and women’s programs] had a meeting with their program managers and it was pretty much stated, you know, “policies are coming out, we are going vape free,” and that’s when their worries and what not came to light. Then the executive team decided to meet with the clients to discuss it further and then probably a week later…clients were like, “well it is in place so we are not going to fight or tell you guys anything, that’s fine.” (P6, 18 months)

Another interviewee conducted focus groups with patients to help shape their implementation plan. These focus groups helped program staff to prepare for the transition by ensuring there were enough alternative activities to avoid unstructured time which the patients felt would increase their cravings:

We did focus groups with clients that were in residence at that time, to discuss the initiative with them and get their feedback on what would make it easier for clients – are we missing anything that we need to do for them, and – things came up – I think the biggest thing that came up from the client perspective was making sure that they didn’t have too much down time, too much free time, where they were left to their own thoughts or devices and likely to go reach for a cigarette, so they wanted to be kept busy. (P5, 12 months)

A third interviewee described a hybrid strategy that included meetings with all patients, as well as a longer-term work group that included patient representatives who could voice concerns and propose strategies for transitioning to tobacco-free grounds:

…With clients we have a work group for this project in particular, we have discussions with clients to get their input before we have meeting with the partner organization…this is where all clients come together. So, I would say our strategy has been to include clients more in advance of implementing the policy to get their input that way we do a better job in trying to get their buy-in. (P3, 12 months)

### Implementation Barriers

#### Cessation Challenges

Cessation challenges, mentioned more often than any other barrier, were described as patients disregarding smoking bans or discontinuing SUD treatment early because they were not allowed to smoke. Several interviewees attributed cessation challenges to the difficulty of stopping nicotine use while dealing with mental health challenges (P11) and other drug use (P6, P9, P11, P15):

… I’ve had clients actually walk out of treatment because they were craving a cigarette. So, it’s – having smoking cessation as part of the treatment process is absolutely vital to someone’s long-term health and sobriety. Because if they’re willing to give up on themselves just to smoke a cigarette, that says a lot right there about their addiction, their level of addiction. And if that’s their level of addiction, then…the chances of us getting them back and getting them back alive is reduced. (P15, 18 months)

Two interviewees (P9, P12) cited the short timeframe of their patients’ treatment stays as a barrier to achieving tobacco cessation:

The program is only 60 days. It’s really short, you know, and they’re still in withdrawal. They still have a lot of things so we can’t ask everything. I’m the counselor here, so I’m working one-on-one with them. It’s -- you can’t get mad at them, you know, to process what they’ve been through and the stress that they are going through right now, you know - any problem and the trauma that they have outside the program - so demanding to stop everything I think is too much, but it doesn’t mean it’s impossible, you know. (P9, 18 months)

Some also mentioned the SUD recovery culture and the need to connect with peers as barriers to tobacco cessation:

The other challenge, I think, is that there’s a lot of tobacco use still in the twelve-step community when they leave. And so that’s something that they’ll be grappling with when they go to those groups. It doesn’t happen in the meeting, but it happens at break, and so that can be triggering for them. (P5, 12 months)

…One of the consistent things I hear from clients is they have a very hard time opening up to others during this time because they’re dealing with so much and losing so much, and one of the things they say helps bond them to others in the group or others that they’re in treatment with is that unity where they can go and smoke. I’ve had some people tell me that they’ve never smoked before, but they will go out and have a cigarette just to be able to talk to other people because it feels more cohesive to them. (P4, 18 months)

While many interviewees emphasized the role of NRT in supporting adherence to smoking bans, one reported that their patients continued to smoke while using the nicotine patch and would “come back sick” from excessive nicotine intake after off-campus Twelve Step meetings or doctors’ appointments (P2, 18 months).

In describing these challenges, some interviewees also reported factors that mitigated patients’ difficulties with the smoking ban. During early implementation, this included turnover so that incoming patients were already aware of the smoking ban, rather than having to adjust to the idea after joining the program:

I would say that for clients that were already here, that were in treatment when we went live with the policy… they were all informed of it when they came in beforehand, so it wasn’t a surprise, but I think they didn’t totally believe that it would happen, or they were thinking it wouldn’t apply to them somehow… they were a little bit more resistant. Now that all of the clients have turned over...it’s been easier. (P5, 12 months)

Several interviewees (P1, P2, P3, P7, P9) noted that total tobacco abstinence seemed easier for patients to achieve than having to time their smoking with off-campus appointments, because the latter situation maintained physical dependence. Two (P1, P2) commented that patients who were admitted directly from jail or prison, where they were not allowed to smoke, seemed to have the easiest time adjusting to the programs’ smoking bans because they were used to not smoking.

#### Staff resistance

Staff resistance to smoking bans manifested as staff continuing to smoke with patients (P4), being reluctant to stop smoking themselves (P2, P5, P8, P9, P11, P12) or enforce smoking bans (P3, P6, P8, P13), and failing to complete required tobacco cessation training (P3, P8). One interviewee commented on how tobacco-free policies may affect the staff’s therapeutic alliance with their patients:

…The other question that came up a lot for staff was…how do I address clients who I find to be sneaking cigarettes, or coming back from medical visits, or whatever, and have gone off-campus and purchased…cigarettes, start confiscating – all this sort of thing, and how to not make it be a negative, punitive, ugly kind of event, or stigmatizing, or diminishing sort of thing. It can feel like going into an incarceration, it can take on that feeling pretty quickly. (P6, 12 months)

Another interviewee echoed this concern, stating that staff’s ambivalence about enforcing the smoking ban sometimes led them to enforce it unevenly, creating additional problems with the therapeutic milieu:

Boundaries are a big issue, both with clients and with staff, and there’s staff that, you know, don’t have the best boundaries and they like to accommodate clients outside of what the policy allows. So I think not only training, but following up with staff and enforcing rules is challenging. And then you end up with someone who is accommodated, and then there’s rumors and miscommunication among clients that needs to be addressed. (P3, 18 months)

A few interviewees attributed staff’s reluctance to enforce smoking bans to feeling like “hypocrites” (P11) for being smokers themselves:

Admittedly we have less staff that smoke now than when we started [the intervention] because they’ve left. But, you know, you bring up the conversation. I’ve got to do a training with staff… and I know that the smokers are gonna roll their eyes, they’re gonna kick their feet. I have told them multiple times, “don't smoke on the front porch.” I go out there and they’re smoking on the front porch, because they don't give a [expletive], and they’re not modeling the behavior that they need to, because they think this is stupid. (P9, 18 months)

Others noted that staff often subscribed to the common idea in SUD recovery culture that smoking cessation should wait until an individual is more stable in their recovery from other drugs:

…There has always been this idea among staff, and I think there still is to a certain extent, that you know, you can’t…it’s applied to nutrition too with the sugary drinks and all that, that you are taking away the vices that they are there to address…so you have to allow them to smoke. They can’t be successful if you take all of their vices away. (P3, 18 months)

Another interviewee (P8) encountered difficulty with nonsmoking staff not understanding that patients were asking for cigarettes because their nicotine cravings needed to be addressed. This interviewee also described repeated incidents in which nursing staff failed to give patients sufficient NRT due to insufficient experience with tobacco cessation treatment:

I think that some of the challenges and the barriers, especially in the beginning, was getting a buy-in from, I guess, the staff at first. … And the nurses did not want to allow them to have the lozenges on them. And they weren’t around, let’s say, to maybe give the next lozenge when you had to suck on them for like one to two hours. So it wasn’t them getting one lozenge a day. They had to have one every one to two hours throughout the day. And so it was failing because I didn’t have people who understood how nicotine replacement therapy worked. (P8, 12 months)

This interviewee resolved the issue by making a “cheat sheet” for the nurses with suggested dosages for different forms of NRT, attending each NRT initiation session to help advocate for the patient’s needs, and then checking the “med book” regularly to ensure patients on NRT were receiving sufficient dosages.

#### Insufficient Capacity

Even with support from the TFR initiative, several interviewees reported shortfalls due to staff turnover (P3, P6, P11, P16), lack of staff expertise in tobacco cessation (P3, P6), and insufficient funds to cover staff positions needed to provide tobacco cessation services (P4, P16), which led to insufficient time for existing staff to provide these services (P4, 16).

Financial concerns were described as the root of capacity shortfalls. One interviewee (P2) recounted that they were initially able to use grant funds to provide NRT to patients during the initiation of the program’s smoking ban, but eventually those ran out and they had to rely on external sources (smokers’ hotlines, medical referrals) with varying success. Another (P3) noted that certain smoking cessation services were “not specifically built into any rates or reimbursement” which caused them to pull funds from other budget lines to cover those services. One interviewee associated the financial struggles with under-funding for SUD treatment services more generally:

…In the SUD treatment world that’s publicly funded, funding is just really, really skimpy, and being able to maintain staff – consistent staffing to provide the needed level of support and expertise is very challenging. So half the battle is being with the clients and keeping them engaged in their treatment plans. But it takes expertise and it takes personnel to do that and we need to be able to pay personnel a decent wage to get trained and to stay and not have to bounce around because of the cost of living and all that. (P5, 12 months)

In a few cases, insufficient funds discouraged programs from implementing smoking bans at all. One interviewee whose program had decided to ban smoking for staff but not for patients stated: “For clients, it’s a fiscal decision and I can’t financially do it, because I don’t need one more reason for guys not to come in here” (P9, 18 months). Another echoed this concern, saying that as a small, private, nonprofit program they could not afford to have a drop in patient census beyond what COVID-19 had already caused (P12, 18 months).

When asked what the most challenging barriers had been during implementation, one interviewee described the compounding effect of short staffing and heavy workloads on insufficient staff expertise in tobacco cessation:

I would say pulling together training opportunities for staff, you know it’s new for staff and therefore it was hard to meet that requirement and to get all of our staff together as they already have a lot of other training they have to do…so finding time again for all of our staff has been challenging given all their other obligations. (P3, 12 months)

## Discussion

Tobacco cessation improves SUD treatment outcomes (Tsoh et al., 2011) and tobacco-free policies in SUD treatment programs are associated with lower smoking prevalence among patients (Gubner et al., 2019; McCuistian et al., 2022). To inform implementation of tobacco-free policies in SUD treatment, we investigated supports and barriers reported by program directors following a tobacco-free policy intervention.

Implementation supports included framing tobacco cessation within a broader wellness context; providing patients with alternatives to smoking (both NRT and leisure activities); seeking community partnerships; gradual change; and patient inclusion.

Other studies have found that framing tobacco cessation interventions within a broader wellness framework may help to reduce nicotine dependence (Gao et al., 2023) and increase motivation to quit among smokers in earlier stages of change (Ziedonis et al., 2007). Physical activity, in particular, may decrease symptoms of anxiety and increase the odds of successful tobacco cessation (Smits et al., 2016). In our sample, gardening was also mentioned repeatedly as a support not only due to stress relief through the activity, but also visually by transforming former smoking spaces into green areas and removing visual smoking cues. A previous study also identified environmental transformations of former smoking spaces into gardens as a support for tobacco-free policies in SUD treatment (Pagano et al., 2016).

Providing alternatives to smoking, specifically NRT and physical activity, was also identified as a support for patient smoking cessation in an SUD workforce intervention (Martinez Leal et al., 2021). In that study, providers helped patients to adopt healthier coping strategies for stress reduction in lieu of tobacco use. Finding healthier coping mechanisms was also identified as an implementation support in a study of a wellness and smoking cessation intervention in group homes for individuals with serious mental illness (Baker et al., 2016). Alternatives to smoking may provide distraction from urges to smoke and may increase motivation by introducing incentives. Previous studies also identified patient incentives as a support for tobacco cessation, including contingency management trials (Secades-Villa et al., 2020) as well as more informal incentives such as additional breaks or privileges (Pagano et al., 2016).

Partnerships between SUD programs and community tobacco-free organizations are seldom mentioned in the literature as a support for tobacco-related interventions in SUD treatment, but strategic partnership building has been a central component of state tobacco control programs (Robbins & Krakow, 2000) and was encouraged as part of the TFR initiative. Although the initiative provided financial and technical assistance, program directors faced challenges covering costs for NRT and staff training in tobacco cessation treatment. To address these challenges, they appealed to other service agencies in their networks, and also sought advice from other programs participating in the TFR initiative. Building community partnerships may support sustainment of healthcare interventions beyond the life of a funding initiative (Scheirer, 2013).

Gradual change was also mentioned as an implementation support, at least during early implementation and for existing patients. Interviewees reported that patients who entered treatment after smoking bans were fully implemented had an easier time adhering to the policies because they knew ahead of time that smoking was not permitted. Incremental change is a central feature of existing tobacco-free intervention models in SUD treatment at the organizational level (Ziedonis et al., 2007).

We found no other studies identifying SUD patient inclusion as a policy implementation support. Patient inclusion has been discussed generally as “service co-production” in healthcare, in which patients participate in shared decisionmaking about health services (Park, 2020). Existing studies of SUD patient inclusion, however, focus on shared decisionmaking between patient and clinician regarding individual medical care (Friedrichs et al., 2016). More research is needed to ascertain the effects of patient inclusion on policy implementation processes and outcomes in SUD treatment.

Implementation barriers included cessation challenges, staff resistance, and insufficient capacity. Cessation challenges, especially related to the social context of smoking among patients in SUD treatment, have been mentioned as a barrier in other studies (Martinez Leal et al., 2021; Matthews et al., 2023; Pagano et al., 2016). Another common subtheme is the use of smoking as a coping mechanism for physiological and emotional stress during SUD recovery. Several studies have identified the need to tailor smoking cessation regimens to individual needs, as some patients may prefer to reduce smoking gradually or wait until post-acute withdrawal symptoms from other substances have resolved to address tobacco use (Martinez Leal et al., 2021), while others may find total tobacco abstinence easier to achieve than reducing cigarettes per day (Iyahen et al., 2023).

As in our study, others have reported that SUD treatment staff may resist implementation of tobacco-free policies due to the belief that discontinuing multiple substances at once may jeopardize recovery (Fokuo et al., 2022) or the perception of tobacco use as less problematic than other substance use (Iyahen et al., 2023). Staff may also have difficulty enforcing smoking bans among patients, especially if they are smokers themselves, which can limit the impact of tobacco-free policies (Eby et al., 2012). Finally, insufficient workforce expertise and financial resources have been identified as an implementation barrier in other studies of tobacco-free policies in SUD treatment (Britton et al., 2023; Fokuo et al., 2022; Knudsen, 2017).

Tobacco cessation during SUD treatment can be supported clinically through non-stigmatizing, clinician-guided “readiness groups” that focus on patients in various stages of change (Guydish et al., 2016). Staff tobacco dependence can be addressed through workforce tobacco cessation resources and incentives (Ziedonis et al., 2006). Training in tobacco cessation approaches can also support clinical staff expertise (Bobo et al., 1997), and financial limitations may be addressed in the short term by drawing on supports such as NRT provided through state-funded “quit lines” (An et al., 2006; Bonevski et al., 2021).

Study findings should be interpreted with caution. The program sample size was relatively small (*N* = 16), although the interview sample size (*N* = 32 across two time frames) was sufficient to reach data saturation, especially given repeated engagement with the interviewees over time (Guest et al., 2006). The data analyzed here were limited to program director interviews, rather than direct observation of actions or behaviors related to policy implementation. Interview data may have been influenced by social desirability bias (Bergen & Labonté, 2019), despite reminders from interviewers that confidentiality would be maintained and that study findings would have no effect on continued or future funding. Also, although we reported data from the “post-intervention” phase of the study, half of our sample had not fully implemented tobacco-free grounds at the time of our 12- and 18-month interviews, which limited our ability to report successful implementation strategies for that policy. Despite limitations, this study is among the first to provide in-depth qualitative data on SUD program directors’ experiences after implementing tobacco-free policies in residential SUD treatment programs.

## Conclusions

Implementation of tobacco-free policies in residential SUD treatment programs is feasible but requires significant planning and support. Single state agencies and other SUD treatment authorities should allocate more financial and technical assistance resources to help ensure effective implementation of tobacco-free policies in SUD treatment programs. These resources may include, for instance, education on how to bill for reimbursement of tobacco cessation services provided (Bloom et al., 2018). Increased dissemination of effective implementation strategies is also instrumental for promoting tobacco-free policies in SUD treatment. Creating evidence-based implementation guidelines, as well as encouraging coalitions or learning communities among SUD treatment programs that have and have not implemented tobacco-free policies, could help to support implementation efforts and promote knowledge exchange.
